# Visfatin and RBP4 gene expression levels in different adipose tissues and insulin resistance

**DOI:** 10.1186/1753-6561-6-S3-P17

**Published:** 2012-06-01

**Authors:** Zeynep Goktas, Shu Wang

**Affiliations:** 1 Nutrition, Hospitality & Retailing Department, Texas Tech University, Lubbock, Texas, 79409, USA

## Background

As an endocrine organ, adipose tissue produces and secretes lots of hormones and adipokines that play important roles in metabolism and inflammation [1]. Varied adipose tissues may affect the inflammation distinctively, mainly because of the different secretion levels of the adipokines. Adipokines like visfatin and retinol binding protein-4 (RBP4) may have important roles in insulin sensitivity and glucose uptake to the cell [2,3]. Our research focuses on different tissue secretions of visfatin and RBP4 and their inflammatory properties.

## Materials and methods

Potential subjects were informed of the voluntary nature of the study and asked to sign a consent form to participate in the study. Fifteen morbidly obese (BMI ≥40) subjects participated in the study. All patients underwent Roux-en-Y gastric bypass surgery. The subjects had their blood drawn as per the usual treatment protocol before surgery. The subjects also had the following tissue samples taken at the time of the surgery: subcutaneous adipose tissue, omental adipose tissue, and mesenteric adipose tissue. These tissues were immediately washed in Krebs-Ringer phosphate solution and frozen in liquid nitrogen in the operation room. Total RNA was isolated using Qiagen RNeasy Lipid Kit (Qiagen, Valencia, CA) and cDNA was synthesized from RNA using SuperScipt III protocol (Life Technologies, Carlsbad, CA). Real-time quantitative PCR was used to quantify relative gene expression of Visfatin and RBP4. Results were analyzed using Student’s t test with P <0.05 being significant.

## Results

Visfatin level was significantly higher in omental adipose tissue than mesenteric and subcutaneous adipose tissues (p<0.05). There was not any significant difference for visfatin levels between mesenteric and subcutaneous adipose tissue. RBP4 was higher in subcutaneous adipose tissue (p<0.05) and there was not any significant difference between mesenteric and omental adipose tissue. There was not any significant relationship between blood glucose and HbA1c levels and visfatin and RBP4 tissue levels.

## Conclusions

Although both the omental and mesenteric adipose tissues are abdominal fat tissues, they have significantly different visfatin expression levels. The relationship between these adipokines and insulin resistance can be better studied with a larger sample size.
